# Blood and urine biomarkers and myocardial infarction: A 2-sample and multivariate combination of Mendelian randomization

**DOI:** 10.1097/MD.0000000000046146

**Published:** 2026-05-12

**Authors:** Yu Ding, Haoyang Ling, Xiuyan Chen, Yuhua Xie, Yiheng Liu, Zhen Zhou, Meiqi Zhou, Nenggui Xu, Shuai Cui

**Affiliations:** a College of Acupuncture Moxibustion and Massage, Anhui University of Traditional Chinese Medicine, Hefei, Anhui Province, China; b School of Internet, Anhui University, Hefei, Anhui Province, China; c Jieshou Hospital of Traditional Chinese Medicine, Fuyang, China; d Department of Cardiology, The Second Affiliated Hospital of Chongqing Medical University, Chongqing, China; e Anhui Province Key Laboratory of Meridian Viscera Correlation Ship, Hefei, Anhui Province, China; f South China Research Center for Acupuncture and Moxibustion, Medical College of Acupuncture, Moxibustion and Rehabilitation, Guangzhou University of Chinese Medicine, Guangzhou, China.

**Keywords:** blood and urine biomarkers, genetic epidemiology, Mendelian randomization, multivariate Mendelian randomization, myocardial infarction

## Abstract

Myocardial infarction (MI) is one of the most serious cardiovascular diseases in the world. Nevertheless, the majority of diagnostic procedures conducted subsequent to the illness do not provide any means to prevent several risks associated with MI. Blood and urine tests are frequently employed in clinical examinations to detect cardiovascular diseases at an early stage. Mendelian randomization (MR) is commonly employed to explore disease–trait relationships and uncover therapeutic targets. Our goal was to explore the genetic links between 35 blood and urine biomarkers and MI. Blood and urine biomarker MR correlations with MI risk were studied. In version R10, the UK Biobank and Finnish databases included blood and urine marker data and MI data (26,060 cases and 343,079 controls). We performed bidirectional 2-sample MR with 4 methods: inverse variance weighted, MR-Egger, weighted median, and weighted mode. Final causal associations were determined by inverse variance weighted. Sensitivity analyses (heterogeneity, pleiotropy) were conducted. MR-PRESSO and PhenoScanner were used to exclude invalid instruments. We used multivariate MR to filter the most important genes without including other positive genes. To identify positive gene pathways and gene networks that cause MI, we employed GeneMANIA for gene prediction. The findings revealed a positive genetic association between the 8 blood and urine biomarker levels and an elevated risk of MI. There are apolipoprotein B (APOB), glycated hemoglobin, high-density lipoprotein cholesterol, low-density lipoprotein cholesterol, sex hormone-binding globulin, triglycerides, and urate. Moreover, APOB, high-density lipoprotein cholesterol, and low-density lipoprotein cholesterol selectively affect MI through the rejection of other positive gene stems. Finally, APOB and numerous genes strongly impact MI development. APOB collaborates with related genes to regulate plasma lipoprotein particle levels, sterol homeostasis, organization, lipid homeostasis, and remodeling in MI. Our research further reveals the causal relationship between MI and blood/urine biomarkers, providing a new perspective for the prevention, diagnosis, and treatment of MI. Blood and urine marker tests can subsequently be conducted based on these results to detect MI and study the underlying mechanisms linking these metabolites to MI.

## 1. Introduction

Myocardial infarction (MI) refers to the death of heart muscle cells caused by prolonged insufficient blood supply.^[1]^ MI, commonly called a heart attack, is the most severe form of coronary artery disease. This condition is a leading cause of death worldwide.^[2]^ According to the World Health Organization, cardiovascular diseases (including MI) cause about 17.5 million deaths annually, accounting for 31% of all deaths.^[3]^ Epidemiologic investigations have revealed that the incidence of MI increases significantly with age. Although MI poses serious hazards to human health, early diagnosis remains difficult. Consequently, accurate prediction is crucial for improving early prevention and clinical interventions for MI.

Cardiovascular diseases constitute a prominent area of research in which biomarkers have received significant attention. Previous reports have shown that biomarkers such as cardiac troponin are useful for the diagnosis of MI.^[4]^ Moreover, over 80% of acute MI cases are caused by coronary atherosclerosis and luminal thrombus.^[5]^ A analysis evaluated a panel of biomarkers (including blood-based hs-cTnT, NT-proBNP, and urinary C-terminal telopeptide of collagen type I) for risk stratification in MI patients.^[6]^ Compared with single-marker approaches, combined approaches improved the prediction of heart failure and mortality. Coronary atherosclerosis and luminal thrombosis are inextricably linked to lipid transport in the blood. Moreover, routine blood and urine tests are commonly performed at medical appointments, and blood and urine biomarkers are important biomarkers used for clinical diagnosis. ECG is the most popular method for diagnosing MI. A study demonstrated that combining ECG with high-sensitivity cardiac troponin (hs-cTn) testing significantly improved early MI detection rates.^[7]^ Thus, we aimed to investigate the genetic associations between blood and urinary markers and MI. The identification of suitable biomarkers is crucial for the early diagnosis of MI. Several pertinent investigations have revealed that blood biomarkers can be utilized to detect Parkinson disease and Alzheimer disease in their early stages.^[8,9]^ Furthermore, blood and urine biomarkers are also used to help diagnose endometriosis.^[10]^ It has been suggested that blood and urine biomarkers are valuable in the cardiovascular field.^[11]^ Some studies have identified potential plasma protein biomarkers of MI through proteomics, which are often found in the circulation.^[12]^ The screening of prognostic biomarkers in patients with acute MI from classical to modern times has also revealed a number of blood and urine biomarkers that may be associated with MI, such as uric acid.^[13]^ Certainly, certain blood and urine biomarkers have been found in specific areas of cardiovascular medicine. These findings indicate that there is a considerable correlation between the dynamics of various blood and urine markers and cardiovascular disease. Determining blood and urine biomarker level alterations can further clarify their associations with MI, providing a foundation for disease diagnosis and treatment. Currently, there is a lack of extensive research on the correlation between blood and urine biomarkers and MI. However, observational studies are hampered by the difficulty of collecting samples and the need to consider many ethical concerns.

MR employs genetic variants as instrumental variables to mitigate bias caused by confounding and reverse causation, thus providing more robust causal inference than conventional observational studies. Studying long-term exposure effects without real intervention is advantageous, as it offers major benefits while minimizing ethical risks.^[14]^ Therefore, comprehensive studies are necessary to elucidate the causal relationship between blood and urine biomarker levels and MI to improve our understanding of the pathogenesis of MI and predict the risk of MI. We performed an MR analysis of MI and 35 blood and urine biomarkers provided by the UKB. We found that 8 blood and urine markers are strongly genetically associated with MI. Furthermore, after the multivariate Mendelian randomization (MVMR) of positive results was analyzed, the most likely marker related to MI was identified. In addition, we used the MR principle and selected 8 positive results without sensitivity exclusions for gene prediction and analysis. We also explored how these biomarkers might influence MI and their interrelationships.

## 2. Methods

### 2.1. Obtaining ethical permission and determining the study design

MR is based on 3 fundamental assumptions: the genetic variants used as the instrumental variable must show a strong and reliable association with the exposure. Genetic instruments should not be associated with confounding factors. Genetic instruments should influence the outcome only through the exposure, not via alternative pathways.^[15]^ In line with these principles, we used comprehensive GWAS summary data, with informed consent obtained from all participants. We rigorously screened instrumental variables, selecting those with *F*-statistics ≥10 for MR analysis. As only aggregate statistical data were used, no additional ethical approval was required.

### 2.2. Data sources

The study used phenotypic and genotypic data from 35 blood and urine biomarkers acquired from the UKB (Table 1). Frequently measured serum (Category 100080) and urine (Category 100083) biomarkers were included. In addition, the study incorporated comprehensive phenotype and genome-wide genotype data from unrelated individuals. Previous descriptions have provided information about the UKB, such as its geographical regions, recruitment processes, and other characteristics.^[16]^ Information on urine and blood assay categories is available on the UKB website (e.g., https://biobank.ndph.ox.ac.uk/showcase/label.cgi?id=100083). Every participant provided informed consent in the UKB. Authorization to retrieve and examine UKB data was granted for UKB project 46478. The UKB received ethical approval from the NHS National Research Ethics Service (reference 11/NW/0382). We were exempt from seeking further ethical approval from our university for our project because it involved the secondary use of data.^[17]^

**Table 1:** Source and details of exposure.

Traits	Sample size (n)	SNPs (n)	SNPs: *P *< 5 × 10 − 8	Population	PMID
Lipoprotein A	253,570	8859383	180	European	33462484
SHBG	289,010	8859357	264	European	33462484
IGF-1	317,114	8859339	169	European	33462484
Phosphate	291,391	8859339	206	European	33462484
Albumin	291,516	8859336	206	European	33462484
Non-albumin protein	291,516	8859336	202	European	33462484
Total protein	291,516	8859336	182	European	33462484
Vitamin D	304,818	8859332	71	European	33462484
Potassium in urine	309,559	8859331	156	European	33462484
Total bilirubin	317,605	8859328	181	European	33462484
C-reactive protein	318,271	8859326	223	European	33462484
HDL cholesterol	291,830	8859322	294	European	33462484
Apolipoprotein A	290,198	8859321	185	European	33462484
Calcium	291,843	8859319	258	European	33462484
Creatinine in urine	310,240	8859318	288	European	33462484
Cystatin C	318,819	8859309	260	European	33462484
Aspartate aminotransferase	317,763	8859308	101	European	33462484
Urate	318,526	8859308	258	European	33462484
Alanine aminotransferase	318,818	8859307	231	European	33462484
Urea	318,724	8859306	307	European	33462484
Glucose	291,605	8859317	214	European	33462484
AST to ALT ratio	317,687	8859316	11	European	33462484
Triglycerides	318,674	8859315	263	European	33462484
Alkaline phosphatase	318,953	8859314	162	European	33462484
Creatinine	318,800	8859313	231	European	33462484
eGFR	318,800	8859313	115	European	33462484
LDL cholesterol	318,340	8859312	82	European	33462484
Testosterone	289,117	8859311	214	European	33462484
Gamma glutamyltransferase	318,779	8859310	209	European	33462484
Apolipoprotein B	317,412	8859309	220	European	33462484
Cystatin C	318,819	8859309	26	European	33462484
Cholesterol	318,927	8859306	66	European	33462484
Sodium in urine	309,585	8859302	0	European	33462484
Direct bilirubin	271,418	8859292	0	European	33462484
HbA1c	304,659	8859287	0	European	33462484
Microalbumin in urine	95,811	8859273	0	European	33462484

Source and details of exposure. The table summarizes the basic information of blood and urine biomarkers used as exposures in the 2-sample Mendelian randomization (TSMR) analysis, with myocardial infarction (MI) as the outcome. For each trait, the sample size, number of genome-wide significant SNPs (*P* < 5 × 10^–8^), study population, and PubMed ID (PMID) are provided. These details ensure the transparency of the data sources and the reproducibility of the analysis.

AST to ALT = AST/ALT ratio, eGFR = estimated glomerular filtration rate. Created in BioRender Yu Ding (2025) https://BioRender.com/5zhc7ca.

The MI GWAS data were obtained from the FinnGen biobank (DF10 – December 18, 2023) and can be accessed at the following link: https://www.finngen.fi/en. The dataset includes MI records from the Finnish national biobank between 1970 and 2019. The dataset was finalized in December 2022. Moreover, this dataset applied the STRICT criteria to minimize false positives. The method demonstrated about 92% sensitivity. More detailed information can be obtained by visiting the FinnGen biobank.

Furthermore, we selected *F* ≥ 10 of the single-nucleotide polymorphisms (SNPs) as our instrumental variables to ensure the reliability and strength of the outcomes.

### 2.3. MR analysis

In this research, we conducted a 2-sample MR (TSMR) analysis with blood and urine biomarkers and MI. Independent SNPs were identified and clumped within a 10,000 kb window (LD *r*² ≤ 0.01, *P* ≤ 5 × 10⁻⁸) for both biomarkers and MI. We employed the R package “Two Sample MR” version 0.5.6 (University of Bristol, Bristol, United Kingdom) for the MR analysis.^[18]^ We carried out data coordination through the following steps to ensure the comparability of the genetic data on exposure and outcome. First, we matched the common SNPs (based on rsID) in the aggregated data of the exposure and outcome GWASs and then aligned the allele directions. When effect alleles were interchanged (e.g., exposure A/C vs outcome C/A), the outcome beta was reversed. If strand orientation was inconsistent (e.g., A/G vs T/C), alleles were converted to complementary bases (A↔T, C↔G). Ambiguous SNPs (A/T or C/G) were resolved using effect allele frequency. Moreover, we used PhenoScanner to identify the SNPs related to MI and then removed them. Finally, we unified the units of effect values (such as using standard deviation as the unit) and verified that the effect alleles of all the SNPs were completely consistent to eliminate coding bias and ensured the reliability of the subsequent instrumental variable analysis.

The major method we used was the inverse variance weighted (IVW) method.^[17]^ IVW is regarded as the “gold standard” due to its efficiency, simplicity, and suitability for linear causal inference.^[17]^ In addition, we chose MR-Egger,^[19]^ the weighted median,^[20]^ and the weighted mode^[20]^ to validate the results. MR-Egger detects violations of standard IV assumptions and provides sensitivity analysis for robustness.^[19]^ Moreover, the weighted median yields consistent estimates even with many invalid instruments. Finally, the weighted mode presented less bias and lower type-I error rates than the other methods did under the null mode in many situations. We primarily relied on IVW *P* values to determine significance, supported by consistency across the other 3 methods. When the genetic frequency of SNPs varies, it is necessary for the odds ratio (OR) to also fall within the 95% confidence interval (CI).^[21]^ We used the following 4 criteria as grades of *P*val significance: *P* > .05 indicates no statistical significance; *P* < .05 indicates a 95% chance that the result is not due to a random error; *P* < .01 indicates highly substantial statistical evidence, indicating a 99% probability of nonrandom error; and *P* < .001 indicates highly significant statistical evidence, indicating a 99.9% probability of nonrandom error. Moreover, except for *F* > 10, owing to the repeated nature of our calculations, we also utilized the false discovery rate (FDR) approach to compensate for *P*-values.^[22]^ A *P*fdr value <.05 was considered statistically significant. After sensitivity tests and MR-PRESSO, PhenoScanner, etc, were conducted, the relevant confounding SNPs were removed, and a second MR analysis was performed to determine the final results. Finally, the Steiger test and reverse MR were applied to assess causal direction.

### 2.4. Sensitivity analysis

Horizontal pleiotropy refers to the situation where genetic polymorphisms linked to the exposure of interest directly influence the outcome through many pathways that are not the expected exposure. Consequently, we proceeded to perform a series of methods to identify any pleiotropy and evaluate the reliability of the findings. Examples include Cochran *Q* statistic, funnel plots, leave-one-out analysis, and MR-Egger intercept tests. First, horizontal pleiotropy was measured by examining the intercept term obtained from MR-Egger regression. If the *P* value of the MR-Egger regression is <.05, it indicates the presence of horizontal pleiotropy, and we will abandon the result.^[19]^ Moreover, heterogeneity was identified when the *P* value of the Cochran *Q* test was <.05.^[19]^ Horizontal pleiotropy was further assessed by the MR-Egger intercept.^[23]^

### 2.5. MVMR

MVMR is an extension of MR that uses multiple genetic variants associated with different risk factors to simultaneously estimate their causal effects on the outcome.^[24]^ We selected positive TSMR results (*P* ≤ .05, OR within 95% CI, no multiplicity in sensitivity analyses) as exposures in the MVMR analysis. This approach allowed us to estimate the specific effects of each blood and urine biomarker on MI. The “MVMR” R package was used to calculate the direct effects of blood and urine biomarkers on MI while simultaneously accounting for the influence of other blood and urine biomarkers.^[25]^ The IVW method was applied, with *P* ≤ .05 considered statistically significant.^[26]^

### 2.6. Evaluation of MVMR assumptions

Sensitivity analyses included MR-Egger intercept tests (*P* ≥ .05) to assess instrument validity and robustness.^[27]^ We assessed the combined instrument strength in the MVMR framework using the Sanderson–Windmeijer conditional *F* statistic (*F* > 10).^[28]^ When *F*-statistics fell below 10, we additionally applied the weighted median or MR-Egger methods to account for heterogeneity.^[25]^ We also used PhenoScanner to identify SNPs related to MI and remove them. Leave-one-out analysis and MR-PRESSO were applied to detect confounding SNPs, which were removed before re-analysis. In addition, we also incorporated penalized lasso and median estimates to improve stability.^[29]^

### 2.7. Prediction of gene function

GeneMANIA is an algorithm that integrates numerous association networks in real time to predict gene function. It predicts gene function rapidly and accurately, outperforming previous methods.^[30]^ An interconnected system of interactions is established, and the intensity of each interaction is assessed. When there is no interaction, an association weight of 0 is given. However, when there is an interaction, a positive value is awarded to represent the strength of the interaction and the reliability of the discovery.^[31]^ GeneMANIA is a commonly utilized tool for evaluating and interpreting gene expression data, building protein interaction networks, and discovering possible gene functions. For example, GeneMANIA has been applied to map associations between cardiac biomarkers and human genes.^[32]^ We used GeneMANIA to predict gene networks based on blood and urine MR markers, identifying inter-gene interactions that may influence MI. Input consisted of candidate gene lists mapped from significant SNPs (*P* ≤ 5 × 10⁻⁸; OR within 95% CI) obtained in the TSMR/MVMR analyses. GeneMANIA integrates multiple functional association networks, including co-expression, physical and genetic interactions, pathways, protein domain similarity, co-localization, and predicted functional links.^[33]^ When ≥ 6 genes were provided, GeneMANIA automatically optimized network weights to maximize connectivity; when ≤5 genes were used, the GO biological process-based weighting was applied by default.^[3]^ Query parameters were set as follows: organism: *Homo sapiens*, maximum related genes: default 20, network weighting: automatic (hybrid), association score: 0 = no interaction; positive values = higher confidence.^[34]^ The output included gene interaction networks and predicted functional annotations, which were further analyzed to assess whether biomarker-related genes converge on pathways relevant to MI.

## 3. Results

**Figure 1.**
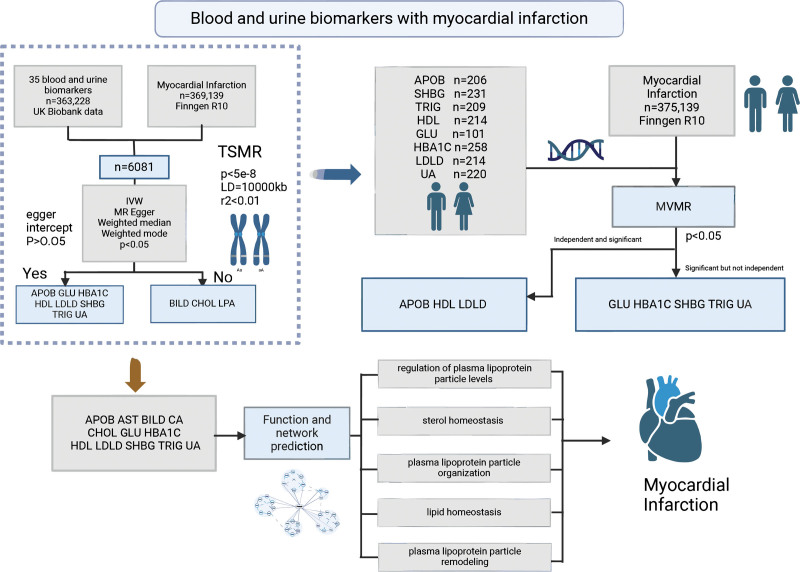
Blood and urine biomarkers with myocardial infarction design flowchart. Data sources UK Biobank (n = 363,228, 35 biomarkers) and FinnGen R10 (n = 369,139, myocardial infarction data); ① 2-sample Mendelian randomization (TSMR) screening: biomarkers identified via inverse variance weighted (IVW), MR-Egger, weighted median/mode methods (*P* < .05) with sensitivity analysis (Egger intercept *P* > .05; SNP filters: *P* < 5 × 10^–8^, linkage disequilibrium (LD) = 100,000 kb, genetic correlation coefficient (*r*^2^) < 0.01), yielding 8 candidates: apolipoprotein B (APOB), glucose (GLU), glycated hemoglobin (HbA1c), high-density lipoprotein cholesterol (HDL), low-density lipoprotein cholesterol (LDL), sex hormone-binding globulin (SHBG), triglycerides (TRIG), urate (UA); ② multivariable Mendelian randomization (MVMR) validation: independent biomarkers (direct causal effects *P* < .05): APOB, HDL, LDL; non-independent biomarkers (associations mediated by confounders): GLU, HbA1c,SHBG, TRIG, UA; ③ functional prediction: Independent biomarkers enriched in lipid homeostasis pathways: regulation of plasma lipoprotein particle levels, sterol homeostasis, plasma lipoprotein particle organization, lipid homeostasis, plasma lipoprotein particle remodeling. Created in BioRender Yu Ding (2025) https://BioRender.com/5zhc7ca.

The MR analysis included 363,228 samples with 35 blood and urine biomarkers from the UK Biobank, as well as 369,139 samples related to MI from the FinnGen R10 study (Fig. 1). The genetic instruments’ minimum *F* value was >10 for all outcome data.

### 3.1. Associations between 35 blood and urine biomarkers and MI in patients with TSMR

For this research, we performed a TSMR analysis using 35 biomarkers found in blood and urine as the factors being studied and MI as the resulting outcome. Summary of the MI analysis results.

**Figure 2.**
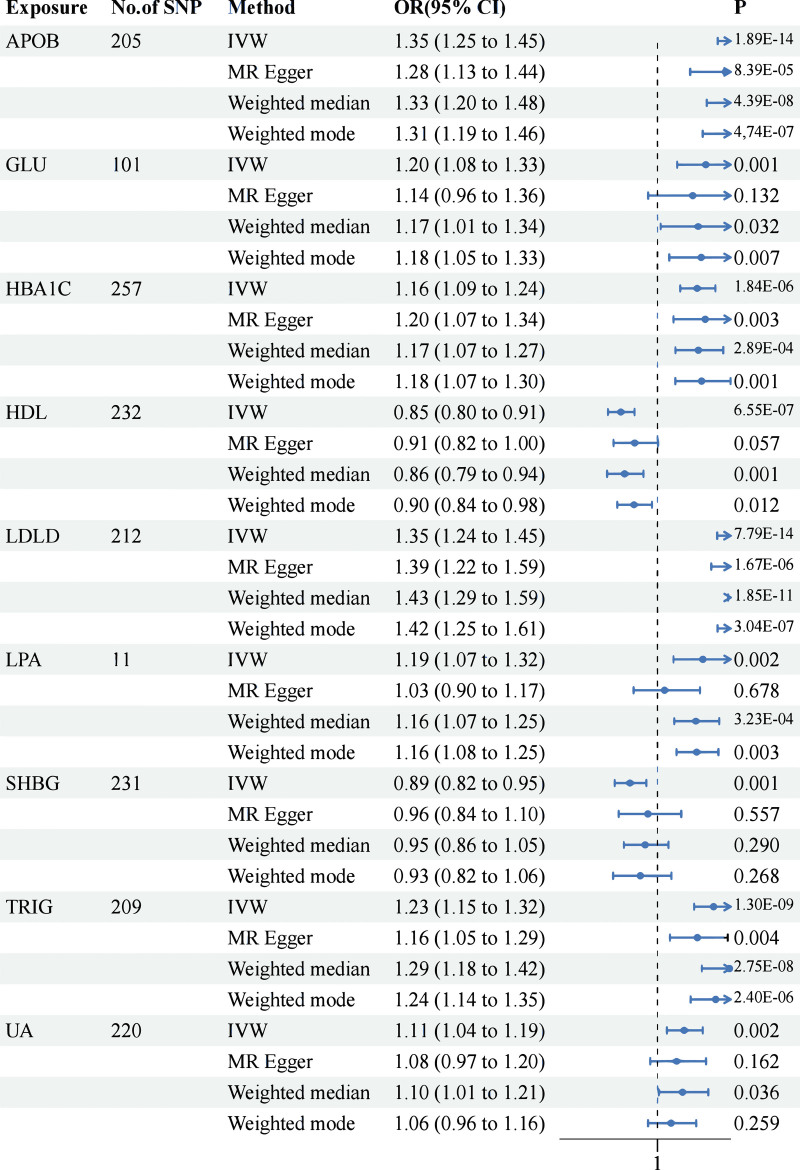
TSMR positive exposure summary forest plot. Two-sample Mendelian randomization (TSMR) analysis was performed to assess the causal effects of blood and urine biomarkers on MI risk. Four complementary methods were applied, including inverse variance weighting (IVW, the primary estimator), MR-Egger regression (sensitive to pleiotropy), weighted median (robust to invalid instruments), and weighted mode. The results indicated that APOB, GLU, HbA1c, LDLD, TRIG, and UA were positively associated with disease risk, whereas HDL and SHBG had protective effects. LPA exhibited a modest risk effect. APOB = apolipoprotein B, GLU = glucose, HbA1c = glycated hemoglobin, LDL = low-density lipoprotein, TRIG = triglycerides, UA = urate. Created in BioRender Yu Ding (2025) https://BioRender.com/5zhc7ca.


By strictly adhering to the screening criteria for instrumental variables in this investigation, a total of 6081 blood and urine biomarkers were ultimately included in the MR analysis. Moreover, we identified the following SNPs related to MI using PhenoScanner: rs34933034, rs1169288, rs10857147, rs35895680, and rs174536. MR-PRESSO et al reported no significant confounding factors. It contains pertinent information on SNPs (Data S1, Supplemental Digital Content, https://links.lww.com/MD/Q793). The study revealed a significant association between genetically predicted levels of 12 blood and urine biomarkers and the risk of MI (*P* < .05) (Fig. 2). The IVW or Wald ratio analysis revealed 11 blood and urine biomarkers, namely, apolipoprotein B (APOB), direct bilirubin (BILD), cholesterol (CHOL), glucose (GLU), HBA1C, HDL cholesterol (HDL), LDL cholesterol (LDLD), lipoprotein A (LPA), sex hormone-binding globulin (SHBG), triglycerides (TRIG), and urate (UA), not only with *P* < .05 but also with a FDR adjusted *P* value of <.05. The ORs and 95% CIs for these associations are as follows:

$$\begin{array}{l} APOB\;(apolipoprotein\;B,\;OR\;=\;1.35,95\;\%\;CI\;\left[1.25,\;1.45\right],\;P\;=1.89\;\times \;10^{\text{\textasciitilde{}}--14}) \end{array}$$

$$\begin{array}{l} BILD\;(Direct\;bilirubin,\;OR\;=\;0.8995\;\%\;CI\;[\;0.83,0.97\;],\;P\;=5.5\;\times \;10^{\text{\textasciitilde{}}--3}) \end{array}$$

$$\begin{array}{l} CHOL\;(cholesterol,\;OR\;=\;1.25\;95\;\%\;CI\;[\;1.16,1.34\;],\;P\;=1.24\;\times \;10^{\text{\textasciitilde{}}--9}) \end{array}$$

$$\begin{array}{l} GLU\;(Glucose,\;OR\;=\;1.20\;95\;\%\;CI\;[\;1.08,1.33\;],\;P\;=5.8\;\times \;10^{\text{\textasciitilde{}}--4}) \end{array}$$

$$\begin{array}{l} HBA1C\;(HBA1C,\;OR\;=\;1.16\;95\;\%\;CI\;[\;1.09,1.24\;],\;P\;=1.84\;\times \;10^{\text{\textasciitilde{}}--6}) \end{array}$$

$$\begin{array}{l} HDL\;(HDL\;cholesterol,OR\;=\;0.85\;95\;\%\;CI\;[\;0.80,0.90\;],\;P\;=6.55\;\times \;10^{\text{\textasciitilde{}}--7}) \end{array}$$

$$\begin{array}{l} LDLD\;(LDL\;cholesterol,\;OR\;=\;1.35,\;95\;\%\;CI\;[\;1.24--1.45\;],\;P\;=7.79\;\times \;10^{\text{\textasciitilde{}}--14}) \end{array}$$

$$\begin{array}{l} SHBG\;(SHBG,\;OR\;=\;0.89\;95\;\%\;CI\;[\;0.82--0.95\;],\;P\;=1.3\;\times \;10^{\text{\textasciitilde{}}--3}) \end{array}$$

$$\begin{array}{l} TRIG\;(triglycerides,\;OR\;=\;1.23,\;95\;\%\;CI\;[\;1.15--1.32\;],\;P\;=1.3\;\times \;10^{\text{\textasciitilde{}}--9}) \end{array}$$

$$\begin{array}{l} UA\;(Urate,OR\;=\;1.1195\;\%\;\left(CI\right)\;1.04--1.19,\;P\;=0.002) \end{array}$$

Based on the MR results, we found that APOB, GLU, HBA1C, TRIG, and UA were genetically positively correlated with MI. However, the MR results negatively correlated HDL and SHBG with MI. The smaller the *P* value in MR is, the greater the statistical significance of the result. The results revealed that APOB, GLU, HBA1C, HDL, LDLD, and TRIG were highly significant (*P* < .001). Moreover, SHBG and UA were highly significantly different (*P* < .01). Moreover, the FDR results are consistent.

### 3.2. Reverse MR and Steiger test

The reverse MR results revealed that among the results that were positive in the forward MR test, all the results except UA were negative. Furthermore, the reverse results of UA are multifaceted, so they need not be taken into account. The results of the Steiger test were all positive, there was no reverse effect (Data S2, supplemental Digital Content, https://links.lww.com/MD/Q793).

### 3.3. Sensitivity analysis for blood and urine biomarkers associated with MI

Moreover, the MR-PRESSO (*P* value for global test > .05), Cochran *Q* test (*P*val > .05), Steiger test (Steiger test = T), and MR-Egger test (Egger intercept = −0.0005, *P* value > .05) results also revealed that APOB, GLU, HBA1C, LDLD, HDL, SHBG, TRIG, and UA had no horizontal pleiotropy. However, some results with *P* > .05 showed horizontal pleiotropy: BILD, CHOL, and LPA. We have discarded these 3 results according to the 3 main principles of MR. Moreover, all of them have heterogeneity. After referring to the large column of examples, we find that even in the presence of heterogeneity in the instrumental variables, the MR analysis provides robust estimates of causal effects, with heterogeneity having a minor impact on the results^[35]^(Table 2). Multivariate MR of positive results for blood and urine biomarkers and MI.

**Table 2:** Summary of the TSMR sensitivity analysis.

Exposure	Outcomes	Cochran *Q* test		MR-Egger	
		*Q* value	*P*	Intercept	*P*
ALB	MI	373.51	8.59	-0.001	.67
ALP	MI	518.29	7.15	-0.003	.05
ALT	MI	369.56	3.49	-0.009	.001
APOA	MI	519.21	3.49	0.006	.01
APOB	MI	490.91	1.71	-0.006	.004
AST	MI	390.35	2.95	-0.006	.02
AST2ALT	MI	418.41	7.58	0.004	.17
BILD	MI	171.34	1.80	0.008	.01
BUN	MI	308.72	2.88	0.004	.24
CA	MI	263.32	5.07E-05	-0.003	.21
CHOL	MI	478.39	7.00	0.001	.72
CRE	MI	504.03	2.19	0.0004	.87
CRP	MI	361.99	1.02	-0.0004	.82
CYS	MI	503.92	3.28	-0.001	.49
EGFR	MI	524.77	3.76	0.0002	.91
GGT	MI	529.42	1.02	-0.003	.18
GLU	MI	177.50	2.95E-06	-0.002	.43
HBA1C	MI	435.45	2.35	0.002	.40

TSMR sensitivity analysis summary. The table presents sensitivity analyses of the associations between blood and urine biomarkers (exposures) and myocardial infarction (MI, outcome) using 2-sample Mendelian randomization (TSMR). Heterogeneity was assessed by Cochran *Q* test (*Q* value and *P* value), whereas directional horizontal pleiotropy was evaluated by the MR-Egger intercept and its *P* value. Non-significant Cochran *Q* values suggest low heterogeneity, whereas a significant MR-Egger intercept indicates potential pleiotropy.

ALB = albumin, ALP = alkaline phosphatase, ALT = alanine aminotransferase, APOA = apolipoprotein A, APOB = apolipoprotein B, AST = aspartate aminotransferase, AST2ALT = AST/ALT ratio, BILD = direct bilirubin, BUN = blood urea nitrogen, CA = calcium, CHOL = cholesterol, CRE = creatinine, CRP = C-reactive protein, CYS = cystatin C, GGT = gamma-glutamyl transferase. Created in BioRender Yu Ding (2025) https://BioRender.com/5zhc7ca.

**Figure 3.**
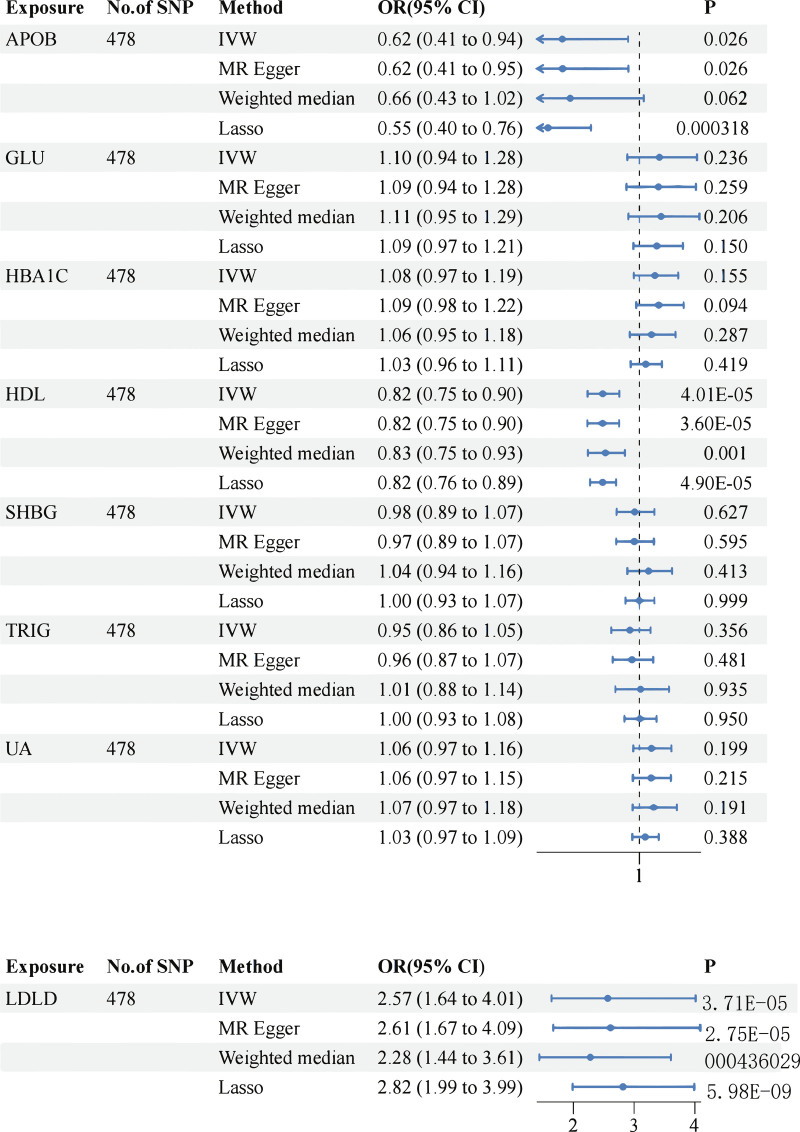
MVMR positive exposure summary forest plot. The forest plot shows the direct effects of blood and urine biomarkers on myocardial infarction (MI) using multivariable Mendelian randomization (MVMR). Four complementary methods were applied: inverse variance weighting (IVW, primary estimator), MR-Egger regression (pleiotropy-robust), weighted median (robust to invalid instruments), and LASSO regression (variable selection under collinearity). Odds ratios (OR) with 95% confidence intervals (CI) are presented. Key findings have shown that LDL cholesterol (LDLD) has a strong and consistent independent positive effect on MI (IVW OR = 2.57, 95% CI [1.64–4.01], *P* = 3.71 × 10^–5^). In contrast, the positive association of apolipoprotein B (APOB) observed in univariable MR disappeared and even reversed after adjustment (IVW OR = 0.62, 95% CI [0.41–0.94], *P* = .026), indicating that there was no independent effect beyond LDLD. HDL cholesterol (HDL) remained inversely associated with MI (IVW OR = 0.82, 95% CI [0.75–0.90], *P* = 4.01 × 10^–5^), whereas other biomarkers, including GLU, HbA1c, SHBG, TRIG, and UA, showed no consistent independent effects. GLU = glucose, HbA1c = glycated hemoglobin, TRIG = triglycerides, UA = urate. Created in BioRender Yu Ding (2025) https://BioRender.com/5zhc7ca.

The interplay of related genes is responsible for the development of numerous illnesses. Thus, we conducted an MVMR study on the positive results of blood and urine biomarkers to identify the most remarkable biomarkers associated with multiple positive genes. The results of our investigation revealed that APOB, high-density lipoprotein (HDL) cholesterol, and low-density lipoprotein (LDL) cholesterol were positive. In the event of a positive result on multivariate analysis and a negative result, the impacts of the genes GLU, glycated hemoglobin (HbA1c), SHBG, triglyceride (TRIG), and UA on MI were not as significant as those of the other genes (Fig. 3). To maintain the accuracy and precision of the findings, we reevaluated the IVW values using the inclusion coefficient penalty approach. The results remained consistent, and the median results were also consistent.

### 3.4. Function and network prediction of MI-associated blood and urine biomarkers

**Figure 4.**
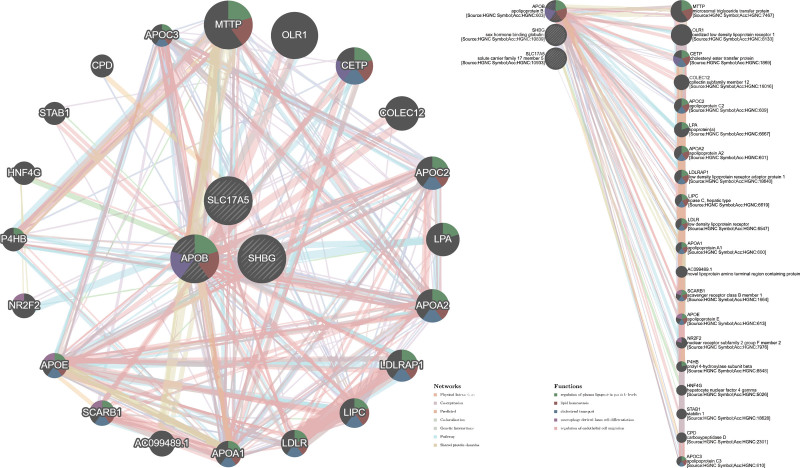
Gene prediction map. The network map illustrates the predicted gene–gene interactions of blood and urine biomarkers associated with myocardial infarction (MI). Key nodes, including APOB, SHBG, CRP, and LPA, are highlighted, with edges representing different types of functional connections: physical interactions, co-expression, predicted interactions, genetic interactions, shared pathways, and protein domains. Node colors indicate functional annotations, such as those related to the regulation of plasma lipoprotein particle levels, protein–lipid complex organization, and plasma lipoprotein particle remodeling. This network emphasizes the central role of lipid-related genes (APOB, APOA2, and LDLR) and inflammatory mediators (CRP and complement components) in mediating cardiometabolic risk. APOA = apolipoprotein A, APOB = apolipoprotein B, CRP = C-reactive protein, GLU = glucose, HbA1c = glycated hemoglobin, LDL = low-density lipoprotein, LPA = lipoprotein A, SHBG = sex hormone-binding globulin. Created in BioRender Yu Ding (2025) https://BioRender.com/5zhc7ca.

Blood and urine biomarkers related to MI have been identified to be associated with networks, specifically in terms of expression and physical interactions (Fig. 4). Moreover, APOB was recognized as a hub for these related genes. Several pathways, including those controlling plasma lipoprotein particle levels and sterol transport, were enriched in cis genes for these blood and urine indicators. Notably, blood and urine biomarkers, such as the regulation of plasma lipoprotein particle levels (FDR: 2.86 × 10^–14^), sterol homeostasis (FDR: 3.79 × 10^–13^), plasma lipoprotein particle organization (FDR: 2.36 × 10^–12^), lipid homeostasis (FDR: 2.58 × 10^–12^), and plasma lipoprotein particle remodeling (FDR: 1.10 × 10^–11^), are crucial contributors to MI.

## 4. Discussion

In this analysis, we identified 8 blood and urine biomarkers associated with MI. APOB, GLU, HbA1c, HDL, LDL, SHBG, TRIG, and UA. In addition, we discovered that APOB, HDL, and LDL show evidence of genetic correlation with MI outcomes when we perform TSMR with positive results from MVMR. LDL cholesterol showed a strong and consistent independent positive effect on MI, HDL remained inversely associated, while the previously positive association of APOB disappeared after mutual adjustment, indicating that there was no independent effect beyond LDL. Other biomarkers (GLU, HbA1c, SHBG, TRIG, and UA) did not show consistent independent effects. All sensitivity tests and directional tests consistently supported the main effect, with no significant level of pleiotropy observed; MVMR *F* ≥ 10.

Blood cholesterol levels, including LDL and HDL, are important indicators for assessing cardiovascular disease risk. High levels of LDL are closely associated with the development of atherosclerosis and cardiovascular diseases. Our TSMR findings and, critically, our MVMR estimates support an independent effect of LDL and an inverse association consistent with an atherogenesis-centered mechanism.^[36]^ Atherosclerosis underlies coronary artery disease, MI, and stroke.^[37]^ Plaque rupture with thrombotic occlusion precipitates MI.^[38]^ However, by facilitating reverse cholesterol transport, HDL is inversely associated with atherosclerotic burden. Therefore, lowering LDL is an established strategy to prevent MI, whereas whether increasing HDL reduces MI risk remains uncertain.^[39]^ Consistent with these results, we provide evidence that LDL and HDL, which are blood and urine biomarkers, can reflect MI. Total CHOL was also positively associated with MI risk. Therefore, HDL is inversely associated with MI; its causal targetability requires further study. In contrast, MVMR indicates a direct, independent effect of LDL on MI. These findings reinforce LDL-lowering as a preventive priority and highlight LDL-related pathways as therapeutic targets for MI.

APOB is the major structural protein of LDL and VLDL, reflecting the total number of atherogenic lipoprotein particles. Elevated APOB levels are strongly associated with atherosclerosis and coronary artery disease, and lowering APOB reduces cardiovascular risk in observational and interventional studies.^[40–42]^ Furthermore, decreasing APOB levels has been found to decrease the probability of acquiring this condition.^[41]^ In our univariable MR analysis, APOB showed a positive genetic association with MI, which is consistent with previous evidence. However, this effect attenuated and reversed in MVMR after adjusting for LDL-C, indicating that APOB does not play an independent causal role beyond LDL but rather serves as a proxy for LDL particle load. Gene network analysis further revealed that APOB is a hub gene that interacts with lipid- and inflammation-related pathways, supporting its biological relevance while underscoring LDL as the central driver of MI pathogenesis.

Our gene prediction and network analysis further support the main findings. The input MR-significant gene set (including APOB, LDLR, C-reactive protein, SHBG, among others) was enriched in lipid metabolism and inflammatory pathways, such as “regulation of plasma lipoprotein particle levels,” “sterol/lipid homeostasis,” and “lipoprotein particle remodeling.” Within the network, APOB and LDLR occupied central hub positions, whereas inflammatory mediators such as C-reactive protein and complement factors formed peripheral interactions, highlighting LDL-centered pathways as critical to MI pathogenesis. We specified the input dataset and parameters (co-expression, pathway, physical, and genetic interactions) and performed random resampling tests, confirming that the observed connectivity was not driven by spurious correlations. Taken together, these mechanistic findings complement our MR evidence and explain why LDL, but not APOB, retained an independent effect on MVMR.

GLU, HAB1C, SHBG, TRIG, and UA were positive in TSMR but negative in the multivariate analysis of positive results. Hyperglycemia, as reflected by GLU and HbA1c, contributes to vascular dysfunction and atherosclerosis.^[43,44]^ SHBG is linked to lipid metabolism and cardiovascular risk, with lower levels associated with higher lipid levels and cardiovascular disease.^[45–47]^ TRIG has been genetically associated with MI, and elevated levels correlate with adverse cardiovascular events.^[48,49]^ Similarly, UA has been implicated in cardiovascular mortality, with higher levels associated with increased MI risk.^[50]^ However, in our MR analyses, these biomarkers were positively associated with MI in the TSMR group, but their effects were no longer significant in the MVMR group, suggesting that they likely act as correlated markers of metabolic or inflammatory dysregulation rather than independent causal factors. These findings highlight the importance of lipid-related biomarkers, such as LDL, as primary causal targets in MI prevention, whereas other biomarkers may serve as secondary indicators.

Sensitivity analyses supported the robustness of our findings. MR-PRESSO (global test *P* ≥ .05), Cochran *Q* test (*P* ≥ .05), the Steiger test (T), and the MR-Egger intercept (-0.0005, *P* ≥ .05) indicated no evidence of horizontal pleiotropy for APOB, GLU, HbA1c, LDL-C, HDL-C, SHBG, TRIG, or UA. Although BILD, CHOL, and LPA showed potential pleiotropy (*P* ≤ .05), these results were discarded according to established MR principles. While some heterogeneity was observed across instrumental variables, prior evidence suggests that moderate heterogeneity does not materially bias causal estimates and that our results remained consistent.^[35]^ Furthermore, reevaluation using the IVW method with penalty adjustment and median-based estimators yielded similar results, reinforcing the reliability of our conclusions.

MR leverages genetic variants as instrumental variables to mitigate confounding and reverse causation, offering stronger causal inference than traditional observational studies.^[51,52]^ Since genetic variants are randomly allocated at conception, MR reduces bias from environmental and behavioral factors and allows assessment of long-term exposure without requiring intervention, thereby minimizing ethical risks.^[52]^ Nonetheless, limitations remain, including potential pleiotropy and restricted generalizability due to the use of European-based samples.

In summary, LDL emerged as the principal independent harmful driver of MI, HDL remained inversely associated, and APOB exhibited a significant inverse direct effect after conditioning LDL in MVMR. This pattern indicates that the APOB primarily reflects the LDL particle number rather than exerting an independent harmful action. Other biomarkers (GLU, HbA1c, SHBG, TRIG, and UA) were associated with the TSMR but not the MVMR, suggesting correlated metabolic or inflammatory states rather than direct causal effects. Notably, well-established diagnostic markers of MI, such as cardiac troponins, were not enriched in our analyses. This absence is expected because troponins mainly rise acutely during myocardial injury, whereas MR captures long-term genetically regulated risk factors, highlighting the complementary roles of genetic epidemiology and clinical diagnostics.

## 5. Conclusion

This study provides robust genetic evidence linking blood and urine biomarkers to MI using MR and MVMR approaches. We identified 8 biomarkers associated with MI risk in the TSMR. In MVMR, LDL retained a strong independent harmful effect, HDL remained inversely associated, and APOB displayed a significant inverse direct effect once LDL was conditioned on, indicating that APOB primarily marks the LDL particle load rather than acting as an independent harmful driver. Notably, established clinical diagnostic biomarkers, such as cardiac troponins, were not enriched in our analyses. This absence is biologically plausible: circulating troponins rise predominantly during acute myocardial injury, whereas the GWAS datasets analyzed here capture steady-state levels of biomarkers in largely healthy populations. Thus, MR can be used to identify long-term genetically regulated risk factors rather than acute damage markers.

Gene prediction and network analysis further highlighted the enrichment of lipid-related pathways and positioned APOB and LDLR as network hubs, supporting a biologically plausible, LDL-centered mechanism. Other biomarkers (GLU, HbA1c, SHBG, TRIG, and UA) were positively associated with the TSMR but not with the MVMR, which is consistent with their roles as correlated indicators of metabolic or inflammatory dysregulation rather than direct causal factors. This distinction underscores the complementary nature of genetic epidemiology and clinical diagnostics: while MR highlights causal pathways in lipid metabolism and inflammation, acute biomarkers such as troponins remain indispensable for the clinical diagnosis and monitoring of MI.

Collectively, these findings reinforce LDL as the primary therapeutic target for MI prevention, suggest complementary roles for HDL in risk reduction, and clarify the role of APOB as a marker of atherogenic particle number with an inverse direct effect conditional on LDL. Integrating univariable and multivariable MR with network analyses provides a framework to prioritize causal targets and refine risk stratification in clinical practice.

## Acknowledgments

We are appreciative of all the participants in this study. Thanks to the providers and collectors of the data used in the text. Thanks to the Biorender website for mapping support. Thanks to all participants and collectors who made the raw data publicly available. We thank the editors and reviewers for their valuable comments on this paper.

## Author contributions

**Conceptualization**: Yu Ding.

**Data curation**: Yu Ding, Haoyang Ling.

**Formal analysis**: Haoyang Ling.

**Funding acquisition**: Meiqi Zhou, Nenggui Xu.

**Investigation**: Xiuyan Chen, Yiheng Liu, Zhen Zhou.

**Methodology**: Xiuyan Chen.

**Software**: Zhen Zhou.

**Validation**: Yuhua Xie.

**Visualization**: Yiheng Liu.

**Writing – original draft**: Yu Ding.

**Writing – review & editing**: Shuai Cui.

## Supplementary Material
